# Splenectomy before adult liver transplantation: a retrospective study

**DOI:** 10.1186/s12893-017-0243-9

**Published:** 2017-04-20

**Authors:** LingXiang Kong, Ming Li, Lei Li, Li Jiang, Jiayin Yang, Lvnan Yan

**Affiliations:** Department of Liver Surgery, West China Hospital of Sichuan University, Chengdu, Sichuan Province China

**Keywords:** Splenectomy, Liver transplantation, Thrombocytopenia, Early allograft dysfunction

## Abstract

**Background:**

A considerable number of patients with portal hypertension (PHT) have to undergo splenectomy because they do not meet the requirements for liver transplantation (LT) or cannot find a suitable liver donor. However, it is not known whether pre-transplantation splenectomy may create occult difficulties for patients who require LT in future.

**Methods:**

We analyzed 1059 consecutive patients who underwent adult liver transplantation (ADLT). Patients with pre-transplantation splenectomy Sp(+) and without splenectomy Sp(−) were compared using a propensity score analysis to create the best match between groups.

**Results:**

There were no differences between patients in group Sp(+) and group Sp(−) with respect to the main post-operative infections (12.20% vs. 15.85%, *P* = 0.455), and the incidence of major complications (6.10% vs. 10.98%, *P* = 0.264). The post-operative platelet count was significantly higher in group Sp(+) (*P* = 0.041), while group Sp(−) had a higher rate of post-operative thrombocytopenia (91.46% vs. 74.39%, *P* = 0.006) and early allograft dysfunction (EAD) (23.20% vs. 10.98%, *P* = 0.038). The 5-year overall survival rates were similar in groups Sp(−) and Sp(+) (69.7% vs. 67.6%, *P* = 0.701).

**Conclusions:**

Compared with Sp(−), the risk of infection and post-operative complications in group Sp(+) was not increased, while group Sp(−) had a higher rate of post-operative EAD. Moreover, pre-transplantation splenectomy is very effective for the prevention of thrombocytopenia after LT. Pre-transplantation splenectomy is recommended in cases with risky PHT patients without appropriate source of liver for LT.

**Electronic supplementary material:**

The online version of this article (doi:10.1186/s12893-017-0243-9) contains supplementary material, which is available to authorized users.

## Background

The incidence of PHT with post-hepatitis cirrhosis is higher in China than elsewhere worldwide [1]. Early complications caused by increased portal pressure, such as variceal hemorrhage, can be treated with vein ligation under endoscopy and injection; however, it is mandatory to switch to decompressive shunt procedures if endoscopic therapy fails to control recurrent variceal hemorrhage [2].

With the advent of LT, simple symptomatic treatment is no longer used for end-stage liver disease, but radical treatment by LT is often performed, greatly increasing the survival rate of patients with end-stage liver disease. Liver diseases are frequently accompanied by PHT, and splenectomy is one of the basic means of treatment of this condition. However, given the great number of patients with end-stage liver disease, a considerable number of patients would require splenectomy to reduce portal pressure, because they do not meet the requirements for transplantation or cannot find suitable liver donor. Splenectomy plays a significant role in improving the survival of patients and can create precious time for subsequent treatment. However, it is not known whether splenectomy may create occult difficulties for patients who require LT in future.

In the past, many scholars stated their views with regard to this point. Starzl et al. [3] were the first to suggest the role of splenectomy in the prolongation of allograft survival, as four of their five patients treated with thymectomy and splenectomy maintained their renal function for almost 6 months. Later, Hume et al. suggested that splenectomy, if performed prior to or at the time of transplantation, could improve leukocyte count and permit administration of large doses of azathioprine (AZA). With a view to preventing thrombocytopenia, and when using AZA after transplantation, splenectomy was often considered as a preventive surgery. With the discovery of cyclosporine and is application after liver transplant, preoperative or intraoperative splenectomy for liver transplant patients has become rather controversial, and is no longer a routine surgery. In addition, Troisi et al. [4] observed that massive ascites loss was associated with extremely high portal flow and histologically proven graft congestion. Splenectomy reduces the portal flow, resolving the ascites problem. However, a relative increase in mesenteric blood flow, containing nutrient-rich blood [5], or a reduction in liver congestion can contribute to liver regeneration [6]. Moreover, patients with hepatitis C virus (HCV) are commonly treated with interferons. Hirotaka et al. [7] have suggested that, to complete pre-emptive interferon therapy, which is initiated approximately 2 months after the operation, splenectomy should be performed simultaneously with LT in HCV patients with a platelet count of < 60/L.

At present, even though there are many related reports, there is no consensus on pre-transplantation splenectomy and current LT standards for hepatocarcinoma (HCC) also lack a description of such patients, such as Milan criteria [8], UCSF criteria [9], Hangzhou criteria [10] and BCLC criteria [11]. Therefore, we studied a series of patients at our institution. We here report the results of a retrospective analysis of adult liver transplant patients who had undergone splenectomy.

## Methods

In our study, the grafts for LT were from donation after cardiac death. No prisoners were included as donors. The protocol was approved by the Ethics Committee of the West China Hospital of Sichuan University West China Hospital. Written informed consent was obtained from all the recipients prior to their surgery, and all of donations were voluntary and altruistic in all cases, and were in accordance with the ethical guidelines of the Declaration of Helsinki.

### Patients

**Fig. 1:**
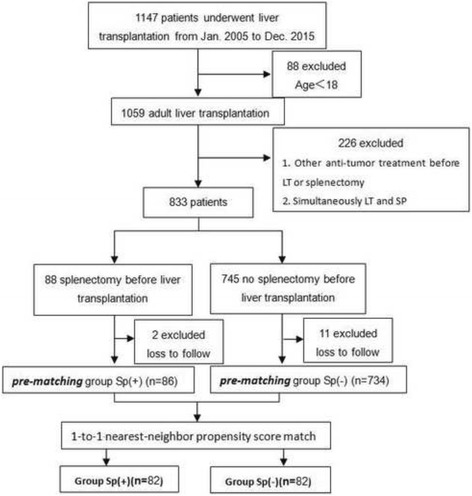
Flow of study participants. Sp(+), Splenectomy before liver transplantation; Sp(−), No splenectomy before liver transplantation

Figure 1 shows the inclusion and exclusion criteria used for establishing the study cohort. Based on the different preoperative intervention methods, they were initially divided into 2 groups: Sp(−)group, which consisted of patients who underwent LT without preoperative splenectomy and the Sp(+), which consisted of those who underwent splenectomy before LT. The indication in our study for splenectomy was progressive, invalidating and/or risky PHT (large splenomegaly, hypersplenism with platelet and/or polymorphonuclear cells count less than 100 Giga/L and 4 Giga/L respectively, grade II or more esophageal varices with the presence of red signs or previous bleeding) in patients with preserved liver and lung function [12]. Disease features and perioperative characteristics of patients when they underwent pre-transplant splenectomy were summarized in Additional file 1: Table S1. All patients were treated with only total splenectomy without partial splenectomy or anther surgery. The indication for LT in our study was end-stage liver diseases [13]. All HCC patients in our study were without vascular invasion and extra hepatic metastasis and summarized in Table 1.

**Table 1:** Baseline demographic and disease features characteristics in the two groups

Variables	Before matching		*P* value	After matching		*P* value
	Sp + (*n* = 86)	Sp- (*n* = 734)		Sp + (*n* = 82)	Sp- (*n* = 82)	
Donor						
Age (mean ± SD, years)	34.38 ± 9.46	33.58 ± 9.62	0.465	34.13 ± 9.39	34.94 ± 9.29	0.582
Male (%)	72(83.3%)	611(82.2%)	0.910	69(84.1%)	67(81.7%)	0.836
height (mean ± SD, cm)	166.62 ± 7.48	167.82 ± 6.69	0.121	166.67 ± 7.45	167.10 ± 6.21	0.689
weight (mean ± SD, kg)	61.56 ± 6.45	63.52 ± 7.38	0.019	62.00 ± 6.29	62.10 ± 6.98	0.913
BMI (mean ± SD, kg/m^2^)	22.14 ± 1.65	22.50 ± 2.05	0.117	22.28 ± 1.53	22.20 ± 1.85	0.779
Recipient						
Age (mean ± SD, years)	46.34 ± 8.44	44.52 ± 9.90	0.102	46.32 ± 8.39	46.41 ± 9.64	0.945
Male (%)	68(79.1%)	619(84.3%)	0.210	64(78.0%)	67(81.7%)	0.697
Height (mean ± SD, cm)	166.90 ± 7.31	167.48 ± 6.43	0.428	166.856 ± 7.40	167.013 ± 7.06	0.889
Weight (mean ± SD, kg)	61.60 ± 9.55	63.42 ± 10.19	0.114	61.67 ± 9.49	61.15 ± 10.07	0.735
BMI (mean ± SD, kg/m^2^)	22.08 ± 2.84	22.56 ± 3.03	0.161	22.11 ± 2.77	21.84 ± 2.81	0.547
HBV (%)	66(76.7%)	574(78.2%)	0.757	62(75.6%)	63(76.8%)	0.854
TB (mean ± SD, μ mol/L)	71.21 ± 98.22	145.73 ± 202.27	0.001	73.54 ± 100.01	83.45 ± 144.73	0.611
ALT (mean ± SD, u/L)	41.54 ± 44.57	29.61 ± 27.63	0.089	36.61 ± 27.09	32.10 ± 22.32	0.363
AST (mean ± SD, u/L)	33.04 ± 35.75	29.65 ± 32.98	0.520	31.81 ± 29.18	29.28 ± 17.80	0.588
Child-Pugh (mean ± SD)	8.71 ± 1.80	8.46 ± 2.13	0.302	8.62 ± 1.76	8.53 ± 1.98	0.751
Child-Pugh <10 (A and B), n (%)	60(69.77%)	531(72.34%)	0.614	58(70.73%)	61(74.39%)	0.600
MELD (mean ± SD)	14.66 ± 6.66	17.54 ± 9.69	0.001	14.82 ± 6.88	14.57 ± 6.50	0.816
Serum AFP > 400 ng/mL (n, %)	14(19.2%)	141(16.3%)	0.511	14(17.1%)	15(18.3)	0.838
Non-tumor (n,%)	53(61.6%)	416(56.7%)	0.380	50(60.97)	49(59.75)	0.873
Number of tumor = 1 (n, %)	27(31.4%)	238(32.4%)	0.395	27(32.9%)	27(32.9%)	1
Number of tumor = 2–3 (n,%)	6(6.98%)	80(10.9%)	0.261	5(6.10%)	6(7.32%)	1
Tumor size < 3 cm (n, %)	25(29.1%)	193(26.3%)	0.581	25(30.5%)	26(31.7%)	0.866
Tumor size > 3 cm (n, %)	8(9.30%)	125(17.0%)	0.080	7(8.54%)	6(7.32%)	0.773
Tumor differentiation grade I, II (n, %)	15(17.4%)	140(19.1%)	0.714	15(18.3%)	14(17.1%)	0.897
Tumor differentiation grade III (n, %)	18(20.9%)	178(24.3)	0.494	17(20.7%)	19(23.2%)	0.706

*BMI* Body mass index, *HBV* Hepatitis B virus, *AFP* alpha-fetoprotein, *MELD* model for end-stage liver disease, *TB* total bilirubin

To ensure the consistency of baseline data, none of the enrolled patients received any other therapies before surgery. All hepatitis B virus (HBV) DNA-positive patients were treated with anti-viral therapy before and after surgery. They were monitored until Dec. 2015 or until their death, and their medical records were retrospectively reviewed. Clinical and demographic data of donors and recipients were collected from the records of the Chinese Liver Transplant Registry (http://cltr.cotr.cn), and patient demographics, disease features, perioperative course, and long-term outcomes were compared between group Sp(+) and group Sp(−) patients.

### Definitions

The Clavien–Dindo complication classification [13] system was used for post-operative complication grading and grade III–IV complications were defined as severe complications. Clinically relevant PHT is defined as the presence of esophageal varices and/or a platelet count of less than 100,000 per mL in association with splenomegaly [14]. EAD defined as the presence of one or more of the following postoperative laboratory: bilirubin ≥10 mg/dL on day 7, international normalized ratio ≥1.6 on day 7, and alanine or aspartate aminotransferases >2000 IU/L within the first 7 days [15].

## Statistical analysis

SPSS 18.0 statistical software (SPSS Inc., Chicago, IL, USA) was used to analyse the relevant data. SPSS 18 did not have a stand-alone function for propensity score analysis, but after the R software and plug-in that could link with the corresponding versions of SPSS and propensity score matching package were installed, propensity score matching could be accomplished by SPSS software [16]. To minimize the influence of other confounders on outcome, we used a propensity score analysis to match Sp(+) patients with Sp(−) patients. Sp(+) patients was matched in a 1:1 ratio with Sp(−) patients using the nearest neighbor matching and based on the variables listed in Table 1. Categorical data were presented as number (per cent) and compared using Pearson chi-Square, Fisher’s exact test. Continuous variables were expressed as the mean value ± SD and analyzed using t-test and repeated measure analysis of variance. Overall patient survival was estimated by the Kaplan–Meier method, and differences between two groups were determined by log-rank test. Independent factors for the platelet count and overall survival were analyzed by multiple regression and Cox proportional hazards models, respectively. *P* < 0.05 was considered statistically significant.

## Results

### Baseline demographic and disease features characteristics

A total of 1147 patients underwent LT between January 2005 and December 2015 at our center. After excluding 11 patients who were lost to follow-up, 734 patients who had not undergone splenectomy before transplantation were retained in pre-matching group Sp(−). After excluding 2 patients who were lost to follow-up, 86 patients who underwent preoperative splenectomy before transplantation were finally included in pre-matching group Sp(+). we used a propensity score analysis to match Sp(+) patients with Sp(−) patients. Sp(+) patients (*n* = 82) was matched in a 1:1 ratio with Sp(−) patients (*n* = 82) using the nearest neighbor matching and based on the variables listed in Table 1.

**Fig. 2:**
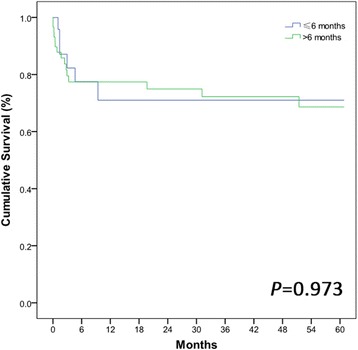
Group Sp(+) are divided into two groups according to the period (≤6 and >6 months) from splenectomy to liver transplantation. There is no significant difference of 1-, 3-, and 5-year OS rates between the two groups (71.0% vs.77.4% *P* = 0.839; 71.0% vs. 72.3%, *P* = 0.992; 71.0% vs. 68.6%, *P* = 0.973)

The baseline characteristics and disease features of Sp(−) and Sp(+) in the pre-match and post-match samples are summarized in Table 1. In the pre-match model, a total of 820 cases were selected to meet the criteria of Sp(+) and Sp(−) group. All cases were 18 years of age or older. In the post-match model, the differences in all variables between Sp(+) and Sp(−) patients were reduced and were therefore not statistically significant. Because period of time after splenectomy may influence the prognosis of LT. We compared the period (≤6 and >6 months) from splenectomy to LT in group Sp(+) (Fig. 2). There is no significant difference of 1-, 3-, and 5-year OS rates between the two groups (71.0% vs.77.4% *P* = 0.839; 71.0% vs. 72.3%, *P* = 0.992; 71.0% vs. 68.6%, *P* = 0.973). The result of single factor analysis with regard to the period from splenectomy to LT related to 5-years OS rates is also not statistically significant (*P* = 0.271).

### Surgical characteristics, post-operative course and survival

Perioperative course post-match samples are summarized in Table 2. There was no significant difference in blood loss during the operation, warm ischemia time and GRWR between group Sp(+) and group Sp(−).

**Table 2:** Perioperative course and postoperative outcome

Variables	Sp + (*n* = 82)	Sp- (*n* = 82)	*P* value
GRWR (mean ± SD)	1.68 ± 0.52	1.65 ± 0.66	0.739
Warm ischemia time (mean ± SD , min)	5.88 ± 6.95	4.66 ± 4.10	0.171
Intraoperative blood loss (mean ± SD , mL)	1478.29 ± 317.40	1571.95 ± 598.78	0.213
Operation time (mean ± SD , hours)	9.30 ± 2.49	9.07 ± 2.16	0.536
Postoperative major complications (Clavien–Dindo ≥ Grade 3) (%)	5(6.10%)	9(10.98%)	0.264
Postoperative infection (%) Mortality (90 days)	10(12.20%) 13(15.9%)	13(15.85%) 8(9.76%)	0.455 0.243
Postoperative portal vein thrombosis (%)	0	0	NS
Postoperative thrombocytopenia (%)	61(74.39%)	75(91.46%)	0.006
EAD (%)	9(10.98%)	19(23.20%)	0.038
Length of postoperative hospital stay (mean ± SD, days)	15.34 ± 1.67	15.76 ± 3.24	0.269

*GRWR* Graft/Recipient’s Body Weight Ratio, *EAD* early allograft dysfunction

**Fig. 3:**
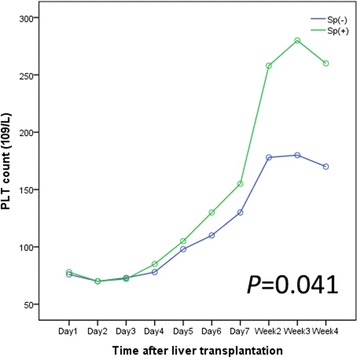
Platelet count changes after liver transplantation

The post-operative course and survival were analyzed using the post-match sample summarized in Table 2. There were no differences between patients in group Sp(+) and group Sp(−) in terms of major infections including lung infection, urinary tract infection, positive blood culture (12.20% vs. 15.85%, *P* = 0.455), the incidence of major complications (Clavien–Dindo ≥ Grade 3; 6.10% vs. 10.98%, *P* = 0.264), and no portal vein thrombosis in both groups. No difference existed between the two groups with regard to 90 days mortality (*P* = 0.243). The length of post-operative hospital stay were similar in group Sp(+) and group Sp(−). In addition, we also analyzed the relationship between group Sp(−) and group Sp(+) with regard to the platelet count and found that group Sp(−) has a higher rate of post-operative thrombocytopenia (91.46% vs. 74.39%, *P* = 0.006), and more EAD than Sp(+) (23.20% vs. 10.98%, *P* = 0.038). The platelet count was significantly higher in group Sp(+) than in group Sp(−) (Fig. 3, *P* = 0.041). Independent factors for the platelet count analyzed by multiple regression were summarized in Table 3. Pre-transplantation splenectomy was considered to be a protective independent factor for post-operative thrombocytopenia. (*P* = 0.002)

**Table 3:** Independent variables in the multiple analysis for postoperative thrombocytopenia (post-matched Sp(+) and Sp(−), *n* = 164)

Variables	Relative risk	*P*
Donor		
Male (%)	−0.550	0.416
Age	−0.003	0.896
BMI	0.171	0.265
Recipient		
Male (%)	0.658	0.352
Age	−0.003	0.896
BMI	0.171	0.265
Splenectomy	−0.1644	0.002
MELD score	0.052	0.275
Child-Pugh score	0.153	0.330
Tumor related	−0.125	0.883
HBV	0.634	0.238
GRWR	0.024	0.965
Warm ischemia time	0.049	0.255
Intraoperative blood loss	−0.001	0.229

**Fig. 4:**
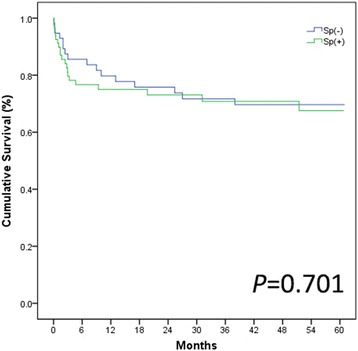
5-year OS rates of the Sp(−) group and the Sp(+) group

The mean follow-up for group Sp(+) was 36.1 months, whereas it was 38.4 months in group Sp(−). Fig. 4 shows that 1-, 3-, and 5-year OS rates of 75.0, 70.8 and 67.6%, respectively, in the Sp(+) group vs. 79.7, 71.7 and 69.7%, respectively, in the Sp(−) group (*P* = 0.459, 0.730,0.701 respectively). Independent factors for overall survival were analyzed by Cox proportional hazards models in Table 4. Tumor related factors were considered independent risk factors for overall survival (*P* = 0.041).

**Table 4:** Independent variables in the Cox analysis for overall survival (post-matched Sp(+) and Sp(−), *n* = 164)

Variables	Relative risk	*P*
Splenectomy	0.191	0.609
Serum AFP	<0.001	0.818
MELD score	−0.038	0.294
Child-Pugh score	0.186	0.157
Tumor related	0.803	0.041
HBV	−0.783	0.054
GRWR	−0.009	0.977
Warm ischemia time	0.006	0.819
Intraoperative blood loss	<0.001	0.478

## Discussion

At present, LT is the last choice for many patients with end-stage liver disease. The wait for transplantation is also long due to the shortage of donor organs. Moreover, LT is not feasible for all patients. Therefore, in order to reduce the pressure on the portal vein, many patients with liver disease choose to undergo splenectomy as a bridging therapy prior to LT. Thus, the influence of splenectomy on LT is still worthy of consideration.

First, the most emphasized risk conveyed by pre-transplantation splenectomy is sepsis. By viewing other relevant literature, on follow-up studies of patients who underwent splenectomy for cirrhosis, but never had a LT, A meta-analysis of follow-up studies involving 19,680 patients showed that the incidence of sepsis among adult splenectomy patients was low, and that a high mortality rate was observed only among children [17]. This meta-analysis thus suggested a low post-operative rate of sepsis after adult splenectomy. Moreover, this is the premise of a low post-operative infection rate in Sp(+) group. Furthermore, for patients who underwent splenectomy and LT synchronously, Samimi et al. [18] reported that synchronous splenectomy resulted in a higher mortality rate, mainly related to septic complications. However, for patients who underwent splenectomy before LT, Jeng et al. [19] and Shimadaet al. [20] found that the risk of infection after LT in patients who had undergone splenectomy remained unchanged. Our results confirm that for the patients who underwent splenectomy before LT (group Sp(+)),the risk of infection was not increased.

We further investigated the influence of splenectomy on post-operative complications after LT, based on the Clavien–Dindo complication classification [21]. 21 deaths occurred in the 90 days after operation in either the Sp(−) or Sp(+) group, and no difference existed between the two groups with regard to various grades of post-operative complications and 90 days mortality. Settmacher et al. [22] reported that concomitant splenectomy during LT was associated with a significant and increased risk of venous complications, such as portal vein thrombosis. However, none of our liver transplant patients in whom splenectomy was performed prior to LT developed portal vein thrombosis. This is consistent with the reports of Hirotaka et al. [7] It is interesting that the incidence of one of the post-operative complications of LT, thrombocytopenia, decreased rather than increased in patients who underwent splenectomy. Many studies have found that the platelet count reaches a nadir at days 2–5 post-transplant, but returned to preoperative levels by weeks 1–2 [23–26]. The reason for this remains unclear. Chang et al. [27] have found that persistent thrombocytopenia is an indicator of a higher rate of fungal infections in liver transplant recipients. On the other hand, platelet-derived serotonin has been found to be important for liver regeneration [28]. A retrospective study [29] has shown that transfused platelets are significantly associated with graft regeneration in liver donors. Furthermore, Bleibel et al. [30] recently observed that peripheral platelet count correlated with liver atrophy and predicted long-term mortality in patients on the liver transplant waiting list. Interestingly, splenectomy can increase the number of platelets, yet portacaval or distal splenorenal shunts cannot increase the number of platelets [31–33]. The reason for this may be that platelets participate in splenic destruction, rather than merely pooling in the spleen [34–36]. In our series of 162 post-matched patients, post-operative peripheral platelet levels were significantly higher (*P* = 0.041) in group Sp(+) than in group Sp(−). Therefore, we believe that splenectomy is an effective approach for increasing platelets. Furthermore, EAD is often used as the best choice of the primary outcome after LT. Li et al. [37] have conducted a retrospective study on adult-to-adult living donor liver transplantation (A-A LDLT) and found that an immediate post-operative platelet count of less than 68 × 10^9^/L was an independent risk factor for post-operative EAD. Interestingly, in our study the Group Sp(−) did have more EAD than Sp(+) (*P* = 0.038). As for its reasons, we considered immediate postoperative low platelet mainly affected the recovery of the liver function by liver regeneration, because there was no significant difference between the two groups with regard to postoperative major complications. The results were also confirmed by us in the living donor liver transplantation [37]. But the specific mechanism still remained to be further studied in the laboratory.

We also compared the overall survival rate of patients between the Sp(−) and Sp(+) groups. In the past, there had been many reports on the effects of LT after splenectomy or simultaneous splenectomy in liver transplant patients, but due to the small number of observations, the results were controversial. At present, there is a lack of a corresponding standard for patients who underwent splenectomy prior to liver transplant. In order to verify the effects of splenectomy on liver transplant patients, we here observed 820 patients in a 5-yearfollow-up study. After analyzing the cumulative overall survival rates of groups Sp(+) and Sp(−), we found no significant differences in the 1-, 3-, and 5-year survival rates in the post-match model. Therefore, we concluded that LT may be suitable for the patients who underwent splenectomy. However, well-designed, long-term, randomized, controlled, prospective trials are still necessary to confirm this proposal.

Through the above analysis, pre-transplantation splenectomy is recommended in cases with risky PHT patients without appropriate source of liver for LT. But one thing to note is that as a “re-operation” the splenectomy is often associated with more difficult dissection due to adhesions. We subjectively believed the transplant operation was perhaps more difficult in the splenectomy group. Although the average time spent on Sp(−) was less than group Sp(+), there was no statistic difference between operative times and intraoperative blood loss. Finally, we believe that adhesion does have some effect on group Sp(+), but it is not obvious.

Our study has several limitations. First, we performed our analysis using only about 82 cases in the Sp(+) group. The limited number of patients may underlie the lack of significant differences and a larger multicenter study should investigate this matter further. Second, these data were retrospectively collected and analyzed, and a prospective clinical investigation should be performed to evaluate the role of splenectomy in this context.

## Conclusions

The results of the present study suggest that pre-transplantation is a very effective method for the treatment of thrombocytopenia after ADLT. Moreover, compared with Sp(−), the risk of infection and post-operative complications in group Sp(+) is not increased, and group Sp(+) had a lower rate of post-operative EAD. There was no difference in the cumulative survival rates between the two groups. Pre-transplantation splenectomy is recommended in cases with risky PHT patients without appropriate source of liver for LT.

## Additional file

Additional file 1: Table S1. Disease Features and Perioperative Characteristics of Splenectomy Patients (*n* = 82). (DOC 34 kb)

## Abbreviations

ADLT: Adult liver transplantation
AFP: Alpha-fetoprotein
AZA: Azathioprine
BCLC: Barcelona Clinic Liver Cancer
BMI: Body mass index
EAD: Early allograft dysfunction
GRWR: Graft/Recipient’s Body Weight Ratio
HBV: Hepatitis B virus
HCC: Hepatocarcinoma
HCV: Hepatitis C virus
LT: Liver transplantation
MELD: Model for end-stage liver disease
PHT: Portal hypertension
Sp: Pre-transplantation splenectomy
TB: Total bilirubin
UCSF: University of California San Francisco
