# Supplementary material for: Splenectomy before adult liver transplantation: a retrospective study
Source: BMC Surg. 2017 Apr 20;17:44. doi: 10.1186/s12893-017-0243-9 (PMC5397796; doi:10.1186/s12893-017-0243-9)
Supplement: Additional file 1: Table S1. — Disease Features and Perioperative Characteristics of Splenectomy Patients (n = 82). (DOC 34 kb) [file 12893_2017_243_MOESM1_ESM.doc]

| **TABLE S1. Disease Features and Perioperative Characteristics of Splenectomy Patients (n=82)** | |
| --- | --- |
| **Variables** | **Value** |
| **Total bilirubin**(mean ±SD,μmol/L) | 43.67±50.31 |
| **ALT**(mean ±SD, u/L) | 41.24±38.10 |
| **AST**(mean ±SD, u/L) | 45.67±51.30 |
| **Child-Pugh A** (%)  **Child-Pugh B** (%)  **Child-Pugh C** (%) | 27(32.93%)  38(46.34%)  17(20.73%) |
| **Tumor related** (%) | 0 |
| **HBV** (%) | 62(75.6%) |
| **Intraoperative blood loss**(mean ±SD , mL) | 319.65±125.23 |
| **Postoperative major complications**  (Clavien–Dindo ≥ Grade 3) (%) | 0 |
